# Outcomes of Phacoemulsification Using Different Size of Clear Corneal Incision in Eyes with Previous Radial Keratotomy

**DOI:** 10.1371/journal.pone.0165474

**Published:** 2016-12-19

**Authors:** Jing Shang Zhang, Xue Liu, Jin Da Wang, Ying Xiong, Jing Li, Xiao Xia Li, Jing Zhao, Qi Sheng You, Yao Huang, Frank Tsai, Larry Baum, Vishal Jhanji, Xiu Hua Wan

**Affiliations:** 1 Beijing Institute of Ophthalmology, Beijing Tongren Eye Center, Beijing Tongren Hospital of Capital Medical University; Beijing Key Laboratory of Ophthalmology & Visual Sciences, Beijing, China; 2 Beijing Tongren Eye Center, Beijing Tongren Hospital of Capital Medical University, Beijing Key Laboratory of Ophthalmology and Visual Sciences, Beijing, China; 3 Jacobs Retina Center, Shiley Eye Institute, University of California, San Diego, California, United States of America; 4 Centre for Genomic Sciences, University of Hong Kong, Hong Kong, China; 5 Department of Ophthalmology and Visual Sciences, the Chinese University of Hong Kong, Hong Kong, China; University of Illinois at Chicago, UNITED STATES

## Abstract

**Objective:**

To evaluate visual outcomes and complications after phacoemulsification in eyes with cataract and previous radial keratotomy (RK) cuts using different sizes of clear corneal incisions.

**Methods:**

The study was a retrospective study. Thirty eyes with cataract and previous RK underwent phacoemulsification and intraocular lens (IOL) implantation. Among them 7 eyes had 8 RK cuts, 13 eyes had 12 RK cuts, and 10 eyes had 16 RK cuts. Phacoemulsification and IOL implantation were performed through a 2.0–3.2 mm clear corneal incision by a single surgeon. In the 8 RK cuts group, 3.2 mm clear corneal incisions were used in 4 eyes, and 3.0 mm clear corneal incisions were used in 3 eyes. In the 12 RK cuts group, 3.2 mm clear corneal incisions were used in 6 eyes, and 2.2 mm clear corneal incisions were used in 7 eyes. In the 16 RK cuts group, 3.2 mm clear corneal incisions were used in 5 eyes, and 2.0 mm clear corneal incisions were used in 5 eyes. Patients were followed up 1 day, 1 week, 1 month, 3 months, 6 months, 1 year, 2 years, and 3 years postoperatively and were examined for the dehiscence of RK cuts during or after the surgery, post-operative best-corrected visual acuity (BCVA), corneal astigmatism, corneal endothelial cell density and complications.

**Results:**

Successful phacoemulsification with IOL implantation was performed in all eyes. No wound dehiscence was noted in any eyes with 8 or 12 RK cuts. Wound dehiscence was noted in 2 eyes with 16 RK cuts. The dehiscence of RK cuts was closed successfully by injecting an air bubble with or without viscoelastic agent into the anterior chamber at the end of surgery. During the follow-up, the cuts were well apposed in all eyes, and no new dehiscence of RK cuts was noted. At the last follow-up, mean BCVA (0.2 ± 0.18 logMAR) was better than preoperative BCVA(0.45±0.19 logMAR) (*P* < 0.001). There was no significant difference between the long-term preoperative and postoperative mean corneal astigmatism (P = 0.3). However, there was a significant reduction in postoperative corneal endothelial cell density (1866.5±773.9 / mm^2^ vs 2421.7±655.7 / mm^2^) (P < 0.001).

**Conclusions:**

Phacoemulsification and IOL implantation with clear corneal incisions in eyes with previous RK were associated with good surgical outcomes. Wound dehiscence was not specificaly related to the size of clear corneal incision during phacoemulsification in these eyes.

## Introduction

Cataract surgery is frequently undertaken in eyes with previous Radial Keratotomy (RK) corneal incisions. It is known that RK cuts inherently weaken the integrity of the cornea [1]. Rabbit models have demonstrated that the amount of force needed for RK incision dehiscence within 90 days after RK surgery is only half of that for the normal eye [2]. RK incision dehiscence after conventional phacoemulsification with clear corneal incision has been reported [3–7]. Our previous studies suggest that phacoemulsification and intraocular lens (IOL) implantation through a 3.2 mm clear corneal incision after 8 or 12 RK cuts is safe, but with risk of dehiscence in eyes with 16 RK cuts [8]. It has been suggested that using a shorter clear corneal incision or a scleral tunnel incision may be safer in the latter case. This current study evaluated the outcomes of phacoemulsification in eyes with cataract and previous RK surgery using clear corneal incisions of different lengths.

## Methods

This was a retrospective study. The study was approved by the Ethics Board of the Beijing Tongren Hospital. The study adhered to the tenets of the Declaration of Helsinki. Written consent was obtained from all the patients before surgery, including the use of their medical data in research. We included consecutive 30 eyes (19 patients) that had a history of RK surgery and that had undergone phacoemulsification through a clear cornea incision for age-related cataract by one surgeon (Wan XH) from January 2011 to August 2015 at the Beijing Tongren Hospital.

### Calculation of the IOL power

The SRK-T formula with manual A-scan and keratometry measurements was used to calculate the IOL power in 10 eyes. The IOL power in the other 20 eyes was calculated by the HOFFER-Q formula with IOLMaster measurements. Among all the 30 eyes, 7 eyes had RK with 8 cuts, 13 eyes had RK with 12 cuts, and 10 eyes had RK with 16 cuts. The type of the IOL was Akreos MI60 (Bausch+Lomb, USA) for the 2.0 and 2.2 clear corneal incisions, the TETRAFLEX (Lenstec, USA) and QUATRIX Aspheric Evolutiv (Croma GmbH, AUSTRIA) IOLs were used for the 3.0 and 3.2 clear corneal incisions.

### Surgical procedure

**Fig 1:**
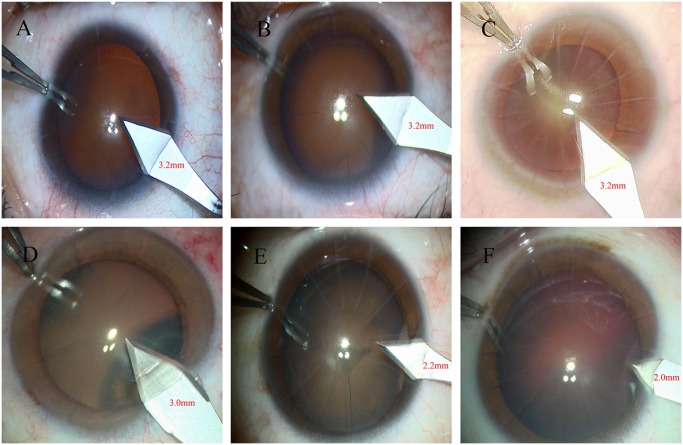
The different sizes of clear corneal incisions for phacoemulsification in eyes with the different RK incisions. (A) The 3.2mm clear corneal incisions in eye with 8 incisions. (B) The 3.2mm clear corneal incisions in eye with 12 incisions. (C) The 3.2mm clear corneal incisions in eye with 16 incisions. (D) The 3.0mm clear corneal incisions in eye with 8 incisions. (E) The 2.2mm clear corneal incisions in eye with 12 incisions. (F) The 2.0mm clear corneal incisions in eye with 16 incisions.

A conventional phacoemulsification with IOL implantation was performed in all eyes using the same phacoemulsification machine and settings (Stellaris, Bausch+Lomb; phacoemulsification; maximum power, 60%; maximum vacuum, 400 mmHg; bottle height, 80 cm). A 3.2 mm keratome was routinely used for clear cornea incision for cataract surgery at our institution before 2012, with recent trend towards a smaller ketatome size (e.g. 2.2 mm) among cataract surgeons. The keratome size was determined at the discretion of the surgeon in current study. In eyes with 8 RK cuts, 4 eyes received phacoemulsification through a 3.2 mm clear corneal incision, and 3 eyes received phacoemulsification through a 3.0 mm clear corneal incision. In eyes with 12 RK cuts, a 3.2 mm incision crossing 1 RK incision was performed in 6 eyes, and a 2.2 mm clear corneal incision was performed without intersecting the adjacent RK incisions in 7 eyes. In eyes with 16 RK cuts, a 3.2 mm clear corneal incision crossed 2 RK incisions in 5 eyes, and a 2.0 mm clear corneal incision was performed without intersecting the adjacent RK incisions in 5 eyes (Fig 1). Subjects were divided into 3 groups for analysis: 8 RK cuts group (7 eyes), 12 RK cuts group (13 eyes) and 16 RK cuts group (10 eyes), Patients were followed up 1 day 1, 1 week, 1 month, 3 months, 6 months, 1 year, 2 years, and 3 years after surgery. Wound dehiscence, complications, postoperative visual acuity, astigmatism, and corneal endothelium cell density were documented. The uncorrected visual acuity was clinically assessed using the Snellen chart, and the best corrected visual acuity (BCVA) was examined by professional optometrists. The Snellen visual acuities were converted to logarithm of the minimum angle of resolution (logMAR) notation, with 0 being the highest score corresponding to 20/20 visual acuity and a value of 1 corresponding to 20/200 visual acuity. The spherical equivalent was examined using the retinoscopy. The corneal astigmatism was calculated (K1-K2) by the pentacam (Oculus, Germany), the corneal endothelial cell density was examined using the corneal endothelial cell counter(SP3000P, Topcon, Japan).

### Statistical analysis

Statistical analysis was performed using SPSS for Windows (version 20.0; IBM-SPSS, Chicago, IL, USA). Descriptive statistics included mean, standard deviation (SD), median, range, and percentages. Paired *t*-test was used to compare preoperative and postoperative BCVA, corneal endothelial cell density, and change of corneal astigmatism stratified in all eyes. Independent sample *t*-test was used to compare the BCVA, corneal endothelial cell density, and change of corneal astigmatism stratified by different corneal incision size in the same cuts group. The variance analysis was used to compare the difference between the SRK-T formula and HOFFER-Q modified formula for the postoperative spherical equivalent and the error between postoperative spherical equivalent and the calculated target refraction. *P*-values represented results for 2-sided tests, with values less than 0.05 considered statistically significant.

## Results

All patients had a cataract grade of nuclear opalescence (NO) 2 or 3 or nuclear color (NC) 2 or 3 according to the Lens Opacities Classification System III (LOCS III) criteria [9]. The mean age of the patients was 53.36 ± 5.53 years (range: 41–61 years). The pre-operative mean BCVA was 0.45 ± 0.19 logMAR (range: 0.2–2). The mean corneal astigmatism was 1.50 ± 0.97 D (range: 0.46 D-3.60 D). The pre-operative mean corneal endothelial cell density was 2421.7±655.7/mm^2^ (range: 730–3370/mm^2^). Overall, 7 eyes had 8 RK cuts, 13 eyes had 12 RK cuts, and 10 eyes had 16 RK cuts. All patients underwent RK between 1992 and 1994. The average time between RK and cataract surgery was 22.68 ± 0.58 years.

The post-operative BCVA was better than pre-operative BCVA. However, the corneal endothelial cell density was significantly lower than the pre-operative corneal endothelial cell density (Table 1). For the corneal astigmatism, there was no significant difference between the 3.2 mm corneal incision group and 3.0 mm corneal incision group in the 8 RK cut eyes (P = 0.338). However, in the 12 and 16 RK cut eyes, the smaller corneal incision eyes had significantly less post-operative corneal astigmatism (P = 0.030, P = 0.041) (Tables 2–4).

**Table 1:** The result of comparing to pre- and post-operative vision, astigmatism, and corneal endothelial cell density of 30 eyes.

Mean age (range) (years)	Variables	Mean BCVA (logMAR)	Mean astigmatism (D)	Mean corneal endothelial cell density (/mm2)
53.36 ± 5.53 (41–61)	Pre-operative	0.45±0.19	1.50±0.97	2421.7±655.7
	Post-operative 1 month	0.15±0.17	1.70±0.69	1953.3±797.6
	P value (compared to pre-operative value)	<0.001	0.51	0.001
	The final visit	0.2 ± 0.18	1.81±0.84	1866.5±773.9
	P value (compared to pre-operative value)	<0.001	0.3	<0.001

**Table 2:** The independent sample t test of pre- and post-operative vision, astigmatism, and corneal endothelial cell density stratified by different corneal incision size in 8 cuts group.

Group	Variables	3.2mm incision group (n = 4 eyes)	3.0mm incision group (n = 3 eyes)	P value
Mean BCVA (logMAR)	Pre-operative	0.72±0.14	0.24±0.15	0.046
	Post-operative 1 month	0.18±0.05	0.05±0.02	0.029
	The final visit	0.14±0.04	0.03±0.01	0.004
Mean astigmatism (D)	Pre-operative	1.72±0.89	1.43±0.58	0.647
	Post-operative 1 month	2.49±0.96	2.00±0.87	0.519
	The final visit	2.69±0.93	2.00±0.72	0.338
Mean corneal endothelial cell density (/mm2)	Pre-operative	2616.83±300.67	2342.93±302.99	0.288
	Post-operative 1 month	2450.76±319.33	1625.90±799.14	0.113
	The final visit	2605.45±432.34	1616.67±800.66	0.086

**Table 3:** The independent sample t test of pre- and post-operative vision, astigmatism, and corneal endothelial cell density stratified by different corneal incision size in 12 cuts group.

Group	Variables	3.2mm incision group (n = 6 eyes)	2.2mm incision group (n = 7 eyes)	P value
Mean BCVA (logMAR)	Pre-operative	0.58±0.36	0.30±0.11	0.070
	Post-operative 1 month	0.27±0.15	0.16±0.08	0.180
	The final visit	0.16±0.09	0.13±0.06	0.745
Mean astigmatism (D)	Pre-operative	1.02±0.78	1.82±1.41	0.245
	Post-operative 1 month	1.82±0.91	1.31±0.63	0.261
	The final visit	2.21±0.85	1.26±0.50	0.030
Mean corneal endothelial cell density (/mm2)	Pre-operative	2514.70±868.26	2630.30±346.61	0.751
	Post-operative 1 month	1688.42±826.22	2363.06±562.99	0.109
	The final visit	1948.27±888.05	2338.19±542.38	0.352

**Table 4:** The independent sample t test of pre- and post-operative vision, astigmatism, and corneal endothelial cell density stratified by different corneal incision size in 16 cuts group.

Group	Variables	3.2mm incision group (n = 5 eyes)	2.0mm incision group (n = 5 eyes)	P value
Mean BCVA (logMAR)	Pre-operative	0.51±0.22	0.40±0.21	0.515
	Post-operative 1 month	0.28±0.11	0.21±0.14	0.463
	The final visit	0.21±0.07	0.17±0.09	0.644
Mean astigmatism (D)	Pre-operative	1.03±0.48	1.46±0.84	0.348
	Post-operative 1 month	2.12±0.45	1.70±0.62	0.230
	The final visit	2.46±0.49	1.54±0.59	0.041
Mean corneal endothelial cell density (/mm2)	Pre-operative	1440.24±868.84	1567.40±764.91	0.789
	Post-operative 1 month	874.60±544.50	1451.58±626.46	0.159
	The final visit	913.56±528.10	1447.80±623.18	0.182

The result of post-operative visual acuity was 0.15±0.17 logMAR at 1st month, 0.14±0.13 logMAR at 3rd month, 0.12±0.09 logMAR at 6th month, 0.13±0.08 logMAR at 12th month. In the 6th month the vision acuity was gradually stabled. The result of post-operative refraction was -0.64±1.27 D at 1 month, -0.54±1.06 D at 3 month, -0.50±1.12 D at 6th month, -0.51±1.03 D at 12th month. Starting from the 6th month the vision acuity and the refraction were gradually stabled. At the 12-month visit, the vision acuity decreased in some patients due to posterior capsule opacification (PCO). These patients were treated with the YAG laser for the PCO.

The data of postoperative refraction was measured and recorded at the last visit. The SRK-T formula and HOFFER-Q modified formula were applied in the study. The results of postoperative spherical equivalent and the error between the actual postoperative spherical equivalent and the target refraction were showed in Table 5. There was no significant difference between the SRK-T formula and HOFFER-Q modified formula for the postoperative spherical equivalent (P = 0.88) and for the error between the actual spherical equivalent and the target refraction (P = 0.71).

**Table 5:** The results of the spherical equivalent and the error between postoperative spherical equivalent and the calculated target refraction by the SRK-T formula and HOFFER-Q modified formula.

	SRK-T formula	HOFFER-Q modified formula
The spherical equivalent (mean±SD)	-0.53±1.05 D	-0.48±1.65 D
The proportion of post operative spherical equivalent within 0±0.5D	45%	41%
The proportion of post operative spherical equivalent within 0±1D	78%	82%
The error between postoperative spherical equivalent and the calculated target refraction (mean±SD)	0.35±0.72 D	-0.31±0.86 D
The proportion of postoperative spherical equivalent within calculated target refraction±0.5D	55%	58%
The proportion of postoperative spherical equivalent within calculated target refraction±1D	84%	88%

**Fig 2:**
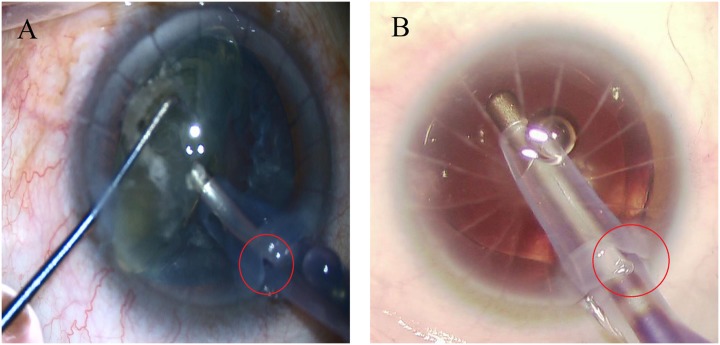
Two eyes with intraoperative RK incision dehiscence. (A) RK wound dehiscence occurred during the process of phacoemulsification. (B) RK wound dehiscence occurred during the process of perfusion aspiration.

In eyes with 8 or 12 RK cuts, there was no intra-operative or post-operative incision dehiscence. Two eyes with 16 RK cuts had intra-operative RK incision dehiscence (Fig 2). One eye of RK wound dehiscence occurred during the process of phacoemulsification, the other eye of RK wound dehiscence occurred during the process of perfusion aspiration. The RK wound dehiscence was not treated during the surgery. Intracameral air bubbles were injected with or without viscoelastic agent in order to maintain the integrity of the anterior chamber at the end of surgery. For all 30 eyes, corneal incisions were water-tight and well apposed at all post-operative visits, and no new RK incision dehiscence was noted.

## Discussion

In previous studies, intraoperative RK incision dehiscence has been reported in cases undergoing clear corneal phacoemulsification [3, 5–7,10]. In these cases, the interval between RK surgery and phacoemulsification ranged from 9 months to 30 years. A 3.0 mm or 3.2 mm clear corneal incision was generally used. RK incision dehiscence mostly occurred intraoperatively during phacoemulsification. In another case report, dehiscence of RK incisions occurred during creation of the main wound [7]. In one case, no dehiscence was noted intraoperatively, but two RK incisions were Seidel positive at post-operative day 1. They were treated with sutures and a bandage contact lens [4].

In our study, the BCVA improved in all patients post-operatively. There was significantly different post-operative astigmatism between different size corneal incision groups in the 12 and 16 RK cut eyes (P = 0.030, P = 0.041). The smaller corneal incision eyes had the less post-operative corneal astigmatism. This finding is similar to another report about the effects of different corneal incisions on postoperative astigmatism in cataract surgery [11]. The location of clear corneal insicion might also affect the post-operative astigmatism. However, in oder to avoid the intersection between the RK cuts and the clear corneal incision, we chose the corneal insicion mainly based on the width between the RK cuts. In many cases, RK incisions may not be uniformly distributed. We recommend that the main incision should be made between two adjacent RK incisions with a comparatively wider space and as peripheral on the cornea as possible, simultaneously considering the convenience of operation to ensure smooth operation. Corneal endothelial cell density declined by different degrees in different groups.

The SRK-T formula and HOFFER-Q modified formula were applied in the current study. Several fomulas have been used to calculate IOL degress for eyes previously underwent refractive surgery, McCarthy et al [12] used the Masket with the Hoffer Q formula, the Shammas.cd with the Shammas-PL formula, the Haigis-L, the Clinical History Method with the Hoffer Q, and the Latkany Flat-K with the SRK-T in cataract surgery of 173 eyes that had uneventful LASIK (89 eyes) or photorefractive keratectomy (84 eyes), the mean arithmetic prediction errors and standard deviations were -0.18±0.87 D, -0.10±1.02 D, -0.26±1.13 D, -0.27±1.04 D, and -0.37±0.91 D, respectively. Finally, they could predict the IOL degree accurately by using these methods, 70% to 85% of eyes could achieve refractive outcomes within 1.0 D of target refraction. Wang et al [13] compared the newer formulae, the optical coherence tomography (OCT)-based intraocular lens (IOL) power formula (OCT formula) and the Barrett True-K formula (True-K), with the methods on the American Society of Cataract and Refractive Surgery (ASCRS) calculator in 104 eyes with previous myopic LASIK/photorefractive keratectomy (PRK). With the OCT, True-K No History, Wang-Koch-Maloney, Shammas, Haigis-L, and Average of these 5 formulas, the percentage of eyes within 0.5 D of refractive prediction error were 68.3%, 58.7%, 50.0%, 52.9%, 55.8%, and 67.3%, respectively, and the percentage of eyes within 1.0 D of refractive prediction error were 92.3%, 90.4%, 86.9%, 88.5%, 90.4%, and 94.2%, respectively. In our study, the formulas could predict the post operative refraction accurately. There was no significant difference between the SRK-T formula and HOFFER-Q modified formula.

In this study, RK incision dehiscence was seen only in 2 eyes that had 16 RK cuts. The gap between adjacent RK incisions in these eyes was narrow, thus the 3.2 mm corneal incision might easily involve adjacent RK incisions, leading to their dehiscence. In order to avoid RK incision dehiscence, the authors propose that the clear corneal incision size should be selected based on the amount of space between RK incisions, with the width of the main corneal incision being less than the distance between two adjacent RK incisions. Based on theoretical calculations, for patients with 12 or 16 RK cuts, the space between two adjacent RK incisions at the corneal limbus is 3.14 mm and 2.30 mm, respectively (Table 6). Therefore, it is suggested that a clear corneal incision length of 3.2 mm or less should be adopted in patients with 8 or fewer RK cuts, 2.2 mm or less for patients with 12 RK cuts, and 2.0 mm or less for patients with 16 RK cuts. These recommendations are supported by the results of our study.

**Table 6:** Space between two neighboring RK incisions at the cornea limbus (mm).

	with 8 RK cuts	with 12 RK cuts	with 16 RK cuts
Theoretical value of the space between cuts*	4.9	3.14	2.30
Suggested value for main corneal incision size	3.2	2.2	2.0

* For the theoretical value calculation, the corneal diameter is assumed to be 11 mm, with RK cuts length about 4.9mm at the corneal limbus.

The interval between cataract surgery and RK surgery may affect the occurrence of RK incision dehiscence. If the interval between cataract and RK surgeries is short, the radial corneal incisions may not have fully healed. It is suggested that corneal wound healing after RK can take up to 47 months [14–17]. This was not likely to affect our results as the average time between cataract and RK surgery was 20 years in our patients. Additionally, in patients with bilateral RK who underwent cataract surgery in both eyes but had dehiscence only in one eye, there may have been variation in the depth of radial corneal incisions, leading to asymmetric tensile strength between the two eyes of the same patient. This highlights the importance of pre-operative inspection for corneal RK incision depth and stability, as well as properly counseling the patient of potential risks.

In previous reports of dehiscence of RK incisions during phacoemulsification, management was performed mainly by suturing [3, 5–7,10]. In our study, intraoperative RK incision dehiscence in 2 eyes was successfully managed with an intracameral air bubble with or without viscoelastic agent injected into the anterior chamber.

Intracameral viscoelastic use during capsulorhexis and hydrodissection should be performed modestly to avoid excessively high intraocular pressure that may predispose to dehiscence of RK cuts. In addition, the intraocular instruments should be maneuvered with extra care to avoid expanding and distorting the incisions. Postoperative eye drops or oral medication may be prescribed to reduce intraocular pressure in the immediate post-operative period. Even if the surgery goes well, re-examination is always needed 1 day after the surgery to determine if there is delayed RK incision dehiscence [4].

In summary, phacoemulsification with a 3.2 mm clear corneal incision can be used safely in patients with 8 RK incisions. However, a corneal incision of 2.2 mm or less is recommended for patients with 12 RK incisions, and a clear corneal incision of 2.0 mm or less for patients with 16 RK incisions to avoid risk of dehiscence. As expected, smaller clear corneal incisions are associated with less post-operative astigmatism.

## References

[1] Peacock LW, Slade SG, Martiz J, Chuang A, Yee RW. Ocular integrity after refractive procedures. Ophthalmology, 1997,104(7):1079–1083. 9224456 10.1016/s0161-6420(97)30182-1

[2] Larson BC, Kremer FB, Eller AW, Bernardino VB Jr. Quantitated trauma following radial keratotomy in rabbits. Ophthalmology, 1983,90(6):660–667. 6888860 10.1016/s0161-6420(83)34502-4

[3] Hui Song, Xin Tang. Wound dehiscence in phacoemulsification after RK surgery: 1 case. Chinese Journal of Ophthalmology, 2009,45(9):848–849.

[4] Day A, Seward H. Delayed radial keratotomy dehiscence following uneventful phacoemulsification cataract surgery. Eye, 2007,21(6):886–887. 10.1038/sj.eye.6702762 17332765

[5] Freeman M, Kumar V, Ramanathan US, O'Neill E.Dehiscence of radial keratotomy incision during phacoemulsification. Eye, 2004,18(1):101–103. 10.1038/sj.eye.6700526 14707986

[6] Behl S, Kothari K. Rupture of a radial keratotomy incision after 11 years during clear corneal phacoemulsification. Journal of cataract and refractive surgery, 2001,27(7):1132–1134. 11489590 10.1016/s0886-3350(01)00763-5

[7] Budak K, Friedman NJ, Koch DD. Dehiscence of a radial keratotomy incision during clear corneal cataract surgery. Journal of cataract and refractive surgery, 1998,24(2):278–280. 9530605 10.1016/s0886-3350(98)80211-3

[8] Xue Liu, Jinda Wang, Jingshang Zhang, Ying Xiong, Jing Li, Xiaoxia Li, et al Efficacy and safety of phacoemulsification with 3.2 mm clear corneal incision for cataract after radial keratotomy. Ophthalmology in China, 2015,24 (6):373–376.

[9] Chylack LT, Wolfe JK, Singer DM, Leske MC, Bullimore MA, Bailey IL, et al The Lens Opacities Classification System III. The Longitudinal Study of Cataract Study Group. Archives of ophthalmology, 1993,111(6):831–836. 8512486 10.1001/archopht.1993.01090060119035

[10] Weili Zhang, Jinying Li. Dehiscence of RK incisions after RK surgery caused by phacoemulsification: 1 case. Chinese Journal of Optometry Ophthalmology and Visual Science, 2015,17(1):60.

[11] Wilczynski M, Supady E, Loba P, Synder A, Palenga-Pydyn D, Omulecki W. Evaluation of surgically induced astigmatism after coaxial phacoemulsification through 1.8 mm microincision and standard phacoemulsification through 2.75 mm incision. Klin Oczna, 2011,113(10–12):314–320. 22384647

[12] McCarthy M, Gavanski GM, Paton KE, Holland SP. Intraocular lens power calculations after myopic laser refractive surgery: a comparison of methods in 173 eyes. Ophthalmology, 2011, 118(5): 940–944. 10.1016/j.ophtha.2010.08.048 21131054

[13] Wang L, Tang M, Huang D, Weikert MP, Koch DD. Comparison of Newer Intraocular Lens Power Calculation Methods for Eyes after Corneal Refractive Surgery. Ophthalmology, 2015, 122(12): 2443–2449. 10.1016/j.ophtha.2015.08.037 26459996 PMC4658226

[14] Luttrull JK, Jester JV, Smith RE. The effect of radial keratotomy on ocular integrity in an animal model. Archives of ophthalmology, 1982,100(2):319–320. 7065953 10.1001/archopht.1982.01030030321020

[15] Waring GO 3rd, Steinberg EB, Wilson LA. Slit-lamp microscopic appearance of corneal wound healing after radial keratotomy. American journal of ophthalmology, 1985,100(1):218–224. 4014376 10.1016/s0002-9394(14)75010-x

[16] Fyodorov SN, Durnev VV. Operation of dosaged dissection of corneal circular ligament in cases of myopia of mild degree. Annals of ophthalmology, 1979,11(12):1885–1890. 556142

[17] Binder PS, Nayak SK, Deg JK, Zavala EY, Sugar J. An ultrastructural and histochemical study of long-term wound healing after radial keratotomy. American journal of ophthalmology, 1987,103(3 Pt 2):432–440. 3826260 10.1016/s0002-9394(14)77767-0

